# Clinical outcomes and safety of finerenone in patients with heart failure: a real-world observational study in China

**DOI:** 10.3389/fcvm.2026.1982954

**Published:** 2026-10-26

**Authors:** Cuimei Zhao, Bing Sun, Huifeng Xu, Yi’an Yao, Feifei Huang, Xuebo Liu

**Affiliations:** Department of Cardiology, Tongji Hospital, School of Medicine, Tongji University, Shanghai, China

**Keywords:** finerenone, heart failure, HFPEF, HFREF, NT-pro BNP

## Abstract

**Objective:**

Finerenone is a highly selective nonsteroidal mineralocorticoid receptor antagonist, but data on its use in patients with heart failure (HF) in real-world clinical practice remain limited. This study evaluated longitudinal clinical changes and safety outcomes observed during finerenone treatment in patients with HF.

**Methods:**

This single-center, retrospective observational study included consecutive patients diagnosed with HF and treated with add-on finerenone between August 2023 and May 2024 at Tongji Hospital, Shanghai, China. All patients received finerenone add-on to standard treatment. Subgroup analyses were performed in patients with HF with preserved ejection fraction (HFpEF), HF with mildly reduced ejection fraction (HFmrEF), and HF with reduced ejection fraction (HFrEF). Adverse events were recorded, including HF readmission, outpatient visits for acute HF, and diuretic dosage escalation. Laboratory and cardiac function markers were also collected.

**Results:**

Seventy-three patients were included. Favorable longitudinal changes were observed in cardiac structure, diastolic function, biomarker levels, and symptom status during follow-up. Left atrial diameter decreased from 50 to 48 mm, the E/e’ ratio decreased from 10.65 to 8.20, and NT-proBNP decreased by 67.47% at 12 months (*P* < 0.001). NYHA functional class distribution also shifted toward lower symptom burden over time, and LVEF showed a modest increase. No patients discontinued finerenone because of adverse events.

**Conclusion:**

In real-world clinical practice, finerenone was associated with a favorable clinical and safety profile in patients with HF, with profiles similar across the HFrEF, HFmrEF, and HFpEF subgroups. Future larger-sample, multicenter randomized controlled trials are warranted to validate these observations.

## Introduction

Heart failure (HF) is the severe terminal stage of various cardiovascular diseases and has become a major global public health issue. The prevalence rate of heart failure among Chinese aged ≥35 is 1.3%, representing over 10 million patients. Furthermore, prevalence increases with population aging (1). Despite recent significant progress in drug therapy [such as angiotensin receptor-neprilysin inhibitors (ARNIs), sodium-glucose cotransporter 2 (SGLT2) inhibitors, etc.] and device therapy, the prognosis of patients with HF remains poor, with a 5-year survival rate of only about 50%. The readmission and mortality rates of HF remain high, imposing a heavy burden on the healthcare system (2, 3). The first-generation steroidal mineralocorticoid receptor antagonist (MRA), spironolactone, and the second-generation MRA, eplerenone, significantly reduce mortality and the risk of hospitalization in patients with HF with reduced ejection fraction (HFrEF) (4, 5), as supported by guidelines (6, 7). However, the efficacy of such drugs in patients with HF with mildly reduced ejection fraction (HFmrEF) and HF with preserved ejection fraction (HFpEF) remains unclear, as revealed by the TOPCAT study despite its criticisms (8), leading to guidelines' conservative attitude towards MRAs in patients with HFmrEF/HFpEF (6, 7).

Finerenone is a recent nonsteroidal MRA approved for the treatment of chronic kidney disease associated with type 2 diabetes in 2021 in the United States of America (USA) and in 2022 by the European Union and China. Compared with traditional steroidal MRAs, finerenone has higher receptor selectivity and different physicochemical properties, with a more balanced distribution in the heart and kidneys (9). Finerenone has significant cardiorenal protective effects in patients with chronic kidney disease complicated with type 2 diabetes. The FIDELIO-DKD and FIGARO-DKD trials confirmed that finerenone can reduce the urinary albumin-to-creatinine ratio, delay the progression of renal function loss, and significantly reduce new-onset HF events (10, 11). Prespecified subgroup analysis showed that finerenone can bring consistent clinical benefits regardless of whether patients have concurrent HF.

The FINEARTS-HF study, published in 2024, showed that in patients with HF and left ventricular ejection fraction (LVEF) ≥40%, finerenone significantly reduced the risk of the composite endpoint of HF worsening and cardiovascular death, and significantly improved the patient-reported Kansas City Cardiomyopathy Questionnaire score (12), providing new hope for the treatment of HFmrEF/HFpEF. Therefore, finerenone was approved in 2025 for the management of HFmrEF and HFpEF in the USA, Canada, and Japan.

However, finerenone's new HF indication is currently supported almost entirely by non-Chinese data, and the National Medical Products Administration (NMPA) of China has not yet approved this indication. A comparison between patients from China and Sweden showed that hospitalized HF patients in China were younger, received less angiotensin-converting enzyme inhibitor (ACEI)/angiotensin receptor blocker (ARB) and β-blocker therapy, and differed in comorbidity profiles compared with Scandinavian/European cohorts, despite having broadly similar short-term prognosis when age-adjusted (13). These phenotypic and treatment differences mean that extrapolating HF trial data (including finerenone trials, largely conducted in Europe/North America) to Chinese patients is uncertain, especially regarding background therapy, age distribution, ischemic burden, and biomarker profiles (14). Although the results of randomized controlled trials are encouraging, data on the application of finerenone in real-world patients with HF remain limited. Real-world studies can reflect the actual efficacy and safety of drugs under conditions of complex comorbidities, polypharmacy, and varying treatment adherence, which are important complements to evidence from randomized controlled trials (15, 16). Given that HF often coexists with comorbidities such as chronic kidney disease and type 2 diabetes, the efficacy and safety of finerenone in real clinical practice urgently need further verification.

Therefore, this study aimed to describe clinical characteristics, longitudinal changes, and safety outcomes among patients with HF receiving finerenone in routine clinical practice. This study provides descriptive real-world data regarding the use of finerenone in Chinese patients with HF.

## Materials and methods

### Study design and population

This single-center, retrospective observational study included consecutive patients diagnosed with HF and treated with add-on finerenone between August 2023 and May 2024 at the Department of Cardiology of Tongji Hospital, Shanghai, China. The study protocol was approved by the Ethics Committee of Tongji Hospital, Shanghai (approval #K-2025-094). The committee waived the requirement for individual informed consent due to the study's retrospective nature.

The inclusion criteria were (1) aged ≥18 years, (2) diagnosed with HF, (3) receiving a standard treatment regimen, based on which finerenone was initiated, and (4) regularly followed up for at least 12 months. The exclusion criteria were (1) concomitant use of potent CYP3A4 inhibitors (e.g., ketoconazole or clarithromycin), (2) baseline serum potassium levels >5.0 mmol/L, (3) baseline estimated glomerular filtration rate (eGFR) <25 mL/min/1.73 m^2^, or (4) acute HF with hemodynamic instability.

### Data collection and outcomes

All data were extracted from the patient charts and clinical databases, including laboratory indicators [serum potassium, serum creatinine (for calculating eGFR), and NT-proBNP levels], cardiac function indicators (New York Heart Association (NYHA) functional class, LVEF, left atrial diameter (LAD), and the ratio between early mitral inflow velocity and mitral annular early diastolic velocity (E/e'), and adverse events.

The observational clinical outcomes were assessed at serial follow-up intervals. NT-proBNP levels and NYHA functional class were evaluated at baseline and at 1, 3, 6, 9, and 12 months. Key echocardiographic parameters—including LAD, E/e', and left LVEF—were measured at baseline, 6 months, and 12 months. Safety outcomes included the incidence of adverse events such as an eGFR decrease >30% from baseline, hyperkalemia (serum potassium >5.5 mmol/L), hypokalemia (serum potassium <3.5 mmol/L), and symptomatic hypotension and clinical events included HF readmission, outpatient visits for acute HF, and diuretic dosage escalation.

Subgroup analyses were performed by HF type. HFrEF was defined as the presence of typical symptoms (e.g., dyspnea and fatigue) and/or signs (e.g., pulmonary rales and lower extremity edema) of HF, with an LVEF of ≤40%. HFmrEF was defined as the presence of symptoms and/or signs of HF, with LVEF between 41% and 49%. A diagnosis of HFpEF had to meet all the following conditions: (1) presence of signs (e.g., jugular vein distension) and/or symptoms (e.g., exertional dyspnea) of HF, or current use of loop diuretics to prevent HF symptoms; (2) LVEF ≥ 50%; (3) objective evidence of left ventricular diastolic dysfunction/elevated filling pressure, with at least one of the following: (i) elevated natriuretic peptide levels: In sinus rhythm, B-type natriuretic peptide (BNP) >35 ng/L and/or N-terminal pro-B-type natriuretic peptide (NT-proBNP) >125 ng/L; for patients with atrial fibrillation, BNP ≥ 105 ng/L or NT-proBNP ≥ 365 ng/L; (ii) abnormal cardiac structure or function confirmed by echocardiography, such as mean E/e' ratio >14 at rest or during exercise stress, or invasive hemodynamic examination showing pulmonary capillary wedge pressure (PCWP) ≥15 mmHg at rest or ≥25 mmHg under stress.

### Statistical analysis

R 4.4.0 was used for statistical analysis. Categorical data were described as *n* (%). Continuous data were tested for normality using the Shapiro–Wilk test. Data conforming to a normal distribution were described as means ± standard deviations, while non-normally distributed data were described as medians (ranges). Intergroup comparisons were performed using independent-samples *t*-tests, analysis of variance (ANOVA), Mann–Whitney *U* tests, or Kruskal–Wallis tests, depending on the data characteristics. Repeated measurement data were analyzed using generalized estimating equations, and pairwise intergroup comparisons were corrected using the Bonferroni method. All tests were two-tailed, with *P* < 0.05 considered statistically significant. Missing data for repeated echocardiographic measurements were not imputed and were handled using available-case analysis. Consequently, subgroup findings with smaller sample sizes are considered exploratory.

## Results

### Characteristics of the patients

Seventy-three HF patients who received finerenone and completed 12 months of follow-up were included in the final analysis (Figure 1). Among them, 25 patients (34.2%) were hospitalized for acute decompensated HF and received intravenous loop diuretics; in these patients, finerenone (10 mg once daily) was initiated prior to discharge once clinical and hemodynamic stabilization was achieved without requiring further escalation of loop diuretic dosing, and a subset of these patients also newly initiated SGLT2 inhibitors during the same hospitalization. The remaining 48 patients (65.8%) were enrolled from the outpatient setting and similarly initiated finerenone at 10 mg once daily. Regarding treatment indications, patients with HFrEF were switched to finerenone due to spironolactone intolerance (e.g., gynecomastia) and the local unavailability of eplerenone, whereas patients with HFmrEF and HFpEF initiated finerenone primarily for comorbid chronic kidney disease and/or type 2 diabetes, or as clinician-directed therapy for persistent HF symptoms.

**Figure 1:**
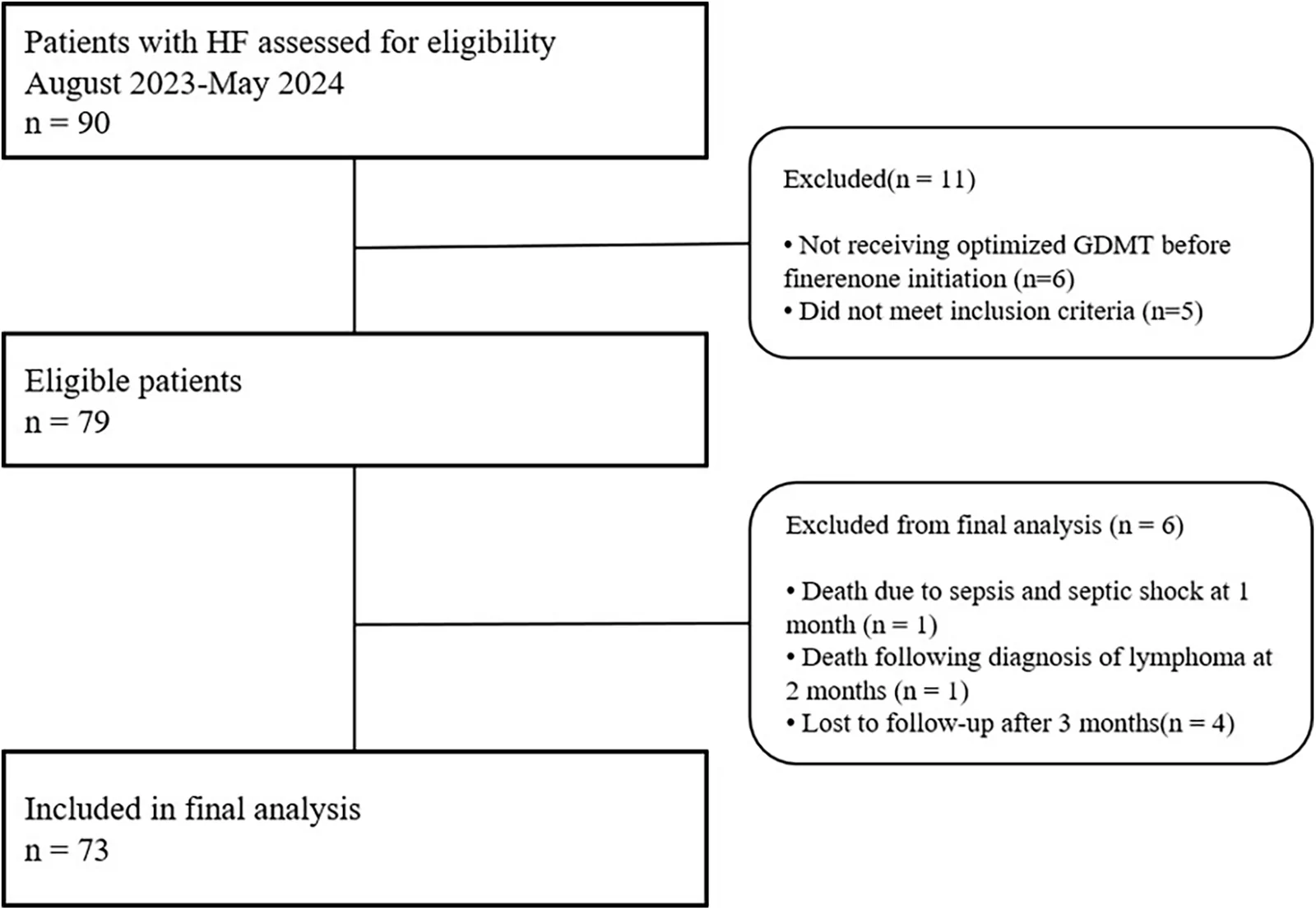
Patient selection and follow-up flow diagram.

The patient characteristics are detailed in Table 1. The median age was 72 years old, and males accounted for the majority (65.8%). The patients had a heavy burden of comorbidities, with coronary heart disease (68.5%), hypertension (65.8%), and type 2 diabetes mellitus (52.1%) being the most common. According to HF classification, HFpEF patients were the main group, accounting for 63.0% (46/73), while HFmrEF and HFrEF patients accounted for 17.8% (13/73) and 19.2% (14/73), respectively. The overall cardiac function was mainly NYHA Class II (58.9%) and Class III (41.1%). The baseline median NT-proBNP level was 1,496.85 pg/mL, indicating that the overall population was at a high risk of HF decompensation.

**Table 1:** Characteristics of the patients.

Parameter	*n*	Total (*n* = 73)
Sex, *n* (%)	73	
Female		25 (34.2)
Male		48 (65.8)
Age, years, median (range)	73	72.00 (41.00, 89.00)
BMI, kg/m^2^, mean ± SD	73	24.74 ± 3.64
eGFR, mL/min·1.73 m^2^, mean ± SD	73	65.39 ± 19.38
eGFR, mL/min·1.73 m^2^, *n* (%)	73	
≤30		2 (2.7)
>30, ≤60		25 (34.3)
>60		46 (63.0)
sK+, mmol/L, mean ± SD	73	3.90 ± 0.45
NT-proBNP, pg/mL, median (range)	73	1,496.85 (331.20, 15,302.00)
NYHA class, *n* (%)	73	
2		43 (58.9)
3		30 (41.1)
LAD, median (range)	73	50.00 (32.00, 76.00)
EF, %, median (range)	73	59.00 (21.00, 71.00)
E/e’, median (range)	62	10.65 (4.60, 24.80)
EF, *n* (%)	73	
HFrEF (EF < 40%)		14 (19.2)
HFmrEF (EF 40%–49%)		13 (17.8)
HFpEF (EF ≥ 50%)		46 (63.0)
SBP, mmHg, mean ± SD	73	127.37 ± 16.47
DBP, mmHg, mean ± SD	73	73.21 ± 10.69
Cr, µmol/L, median (range)	73	92.00 (50.10, 198.10)
UA, µmol/L, median (range)	73	391.00 (222.10, 769.00)
BUN, mg/dL, median (range)	73	7.34 (4.08, 17.80)
TC, mmol/L, median (range)	65	3.61 (1.65, 6.73)
TG, mmol/L, median (range)	65	1.08 (0.41, 3.97)
HDL, mmol/L, median (range)	65	1.09 (0.55, 2.14)
LDL, mmol/L, mean ± SD	65	2.35 ± 0.81
MAU, mg/g, median (range)	44	79.45 (1.48, 2,861.10)
HbA1c, %, median (range)	71	6.80 (5.10, 11.80)
CRP, mg/dL, median (range)	67	2.90 (0.20, 86.30)
Comorbidities, *n* (%)		
Coronary heart disease	73	50 (68.5)
Hypertension	73	48 (65.8)
Type 2 diabetes	73	38 (52.1)
Atrial fibrillation	73	27 (37.0)
Valvular heart disease	73	7 (9.6)
Dilated cardiomyopathy	73	3 (4.1)
Hypertrophic cardiomyopathy	73	4 (5.5)
Concomitant medications, *n* (%)		
ARNI	73	25 (34.2)
ARB	73	38 (52.1)
β-blocker	73	38 (52.1)
sGC	73	3 (4.1)
Lipid-lowering drugs	73	40 (54.8)
Antiplatelet drugs	73	36 (49.3)
Anticoagulants	73	27 (37.0)
Diuretics	73	47 (64.4)
Hypoglycemic drugs	73	44 (60.3)
SGLT2 inhibitor	73	39 (53.4)
GLP-1 receptor agonist	73	1 (1.4)
Metformin	73	11 (15.1)
DPP4 inhibitor	73	3 (4.1)
Insulin and analogs	73	10 (13.7)
AGI	73	5 (6.8)

BMI, body mass index; SD, standard deviation; eGFR, estimated glomerular filtration rate; sK+, serum potassium; NT-proBNP, N-terminal pro-B-type natriuretic peptide; NYHA, New York Heart Association; LAD, left atrial diameter; EF, ejection fraction; HFrEF; heart failure with reduced ejection fraction; HFmrEF, heart failure with midly reduced ejection fraction; HFpEF, heart failure with preserved ejection fraction; SBP, systolic blood pressure; DBP, diastolic blood pressure; Cr, creatinine; UA, uric acid; BUN, blood urea nitrogen; TC, total cholesterol; TG, triglycerides; HDL, high-density lipoprotein; LDL, low-density lipoprotein; MAU, microalbuminuria; HbA1c, glycated hemoglobin; CRP, C-reactive protein; ARNI, angiotensin receptor neprilysin inhibitors; ARB, angiotensin receptor blocker; sGC, soluble guanylate cyclase; SGLT2, sodium-glucose cotransporter-2 inhibitors; GLP-1, glucagon-like peptide-1; DPP4, dipeptidyl peptidase 4; AGI, alpha-glucosidase inhibitors.

In terms of renal function and electrolytes, the baseline average eGFR was 65.39 ± 19.38 mL/min/1.73 m^2^, and 37% of patients (27/73) had chronic kidney disease (eGFR ≤ 60 mL/min/1.73 m^2^). The baseline serum potassium was normal, with an average of 3.90 ± 0.45 mmol/L.

Regarding background regimens, all patients received standard medical therapy tailored to their HF phenotype and comorbidities. Specifically, patients with HFrEF were treated with RASi (ARNI or ARB) and β-blockers alongside finerenone (as a substitute for spironolactone due to intolerance), whereas patients with HFpEF received RASi and other agents primarily for underlying coronary heart disease and hypertension. Across the entire cohort, the combined usage rate of RASi was 86.3%, β-blockers 52.1%, SGLT2 inhibitors 53.4%, and loop diuretics 64.4%. After the initiation of finerenone, aside from appropriate loop diuretic dose adjustments based on volume status and HF symptoms [including dose escalation in seven patients (9.6%)], the drug classes and dosages of all other background medications remained unchanged throughout the 12-month follow-up.

### Clinical and echocardiographic changes

Changes in cardiac structure and diastolic function were observed during follow-up. LAD significantly decreased from 50 to 49 mm at 6 months, and further decreased to 48 mm at 12 months (*P* < 0.001) (Figure 2A and Table 2). The E/e’ ratio, a key indicator reflecting left ventricular filling pressure and diastolic function, significantly decreased from a median value of 10.65–9.20 at 6 months and to 8.20 at 12 months (*P* < 0.001) (Figure 2B and Table 2). Similar trends were observed across the HFrEF, HFmrEF, and HFpEF subgroups (Table 3).

**Figure 2:**
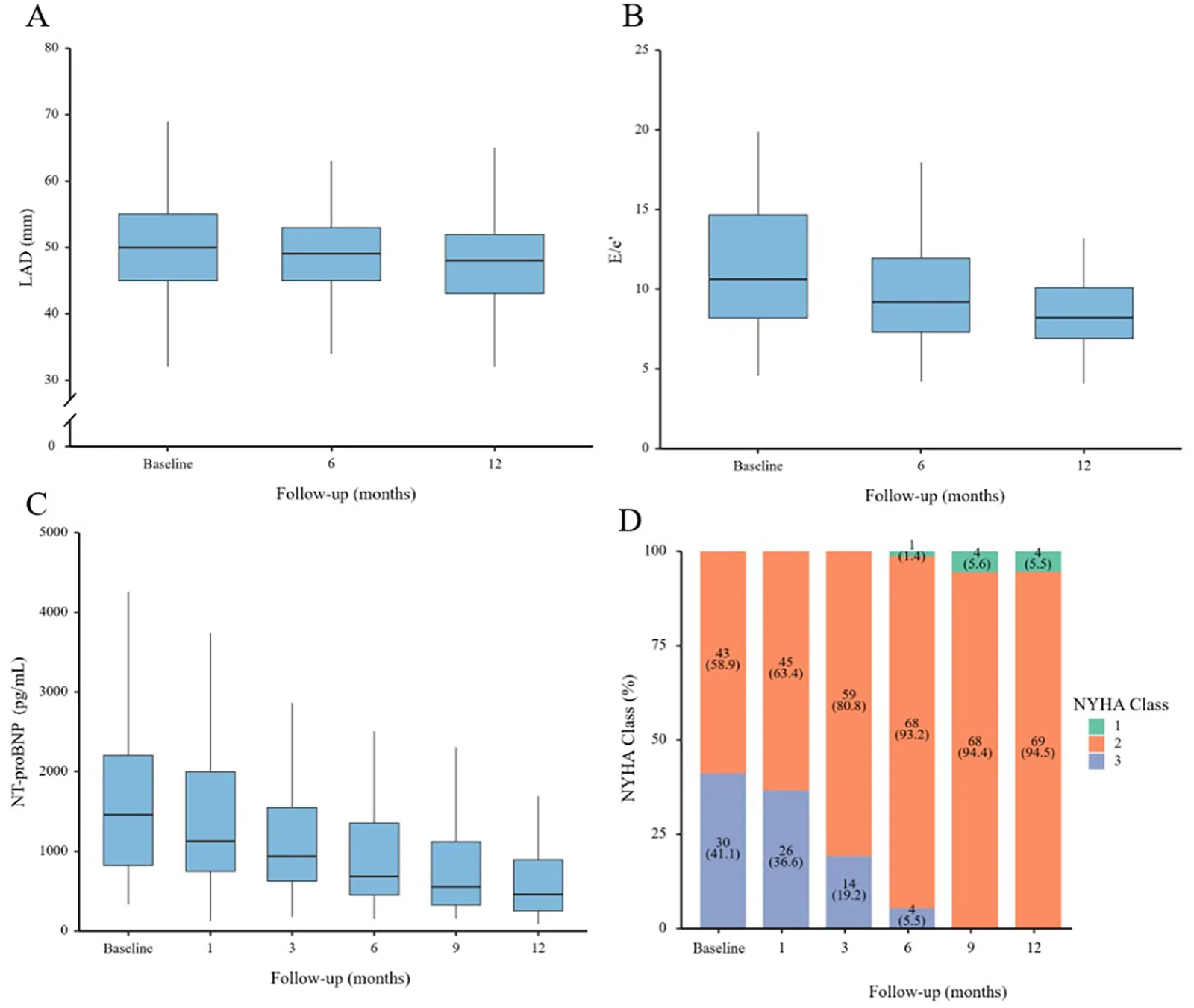
Effects of finerenone on cardiac structure, function, biomarkers, and symptoms in patients with heart failure. **(A)** Change in left atrial anteroposterior diameter. **(B)** Change in E/e’ ratio. **(C)** Change in N-terminal pro-B-type natriuretic peptide (NT-proBNP) levels. **(D)** Change in New York Heart Association (NYHA) functional class distribution.

**Table 2:** Overall effectiveness.

Follow-up	1 month	3 months	6 months	9 months	12 months	*P* *
LAD, mm						
Median (range)			49.00 (31.00, 75.00)		48.00 (32.00, 73.00)	<0.001
Change, median (range)			−1.00 (−11.00, 5.00)		−2.00 (−15.00, 2.00)	
E/e’						
Median (range)			9.20 (4.20, 24.30)		8.20 (4.10, 13.20)	<0.001
Change, median (range)			−0.85 (−1.60, 3.90)		−1.15 (−2.50, −0.30)	
*N* (missing)			62 (11)		63 (10)	
EF, %						
Median (range)			59.00 (30.00, 73.00)		60.00 (30.00, 72.00)	<0.001
Change, median (range)			2.00 (−12.00, 26.00)		3.00 (−12.00, 28.00)	
NT-proBNP, pg/mL						
Median (range)	1,141.50 (120.80, 9,239.00)	945.00 (176.30, 6,308.00)	689.00 (146.50, 7,189.00)	554.30 (149.80, 6,302.00)	457.60 (91.60, 6,573.00)	<0.001
Change, %, median (range)	−12.21 (−24.29, −4.41)	−30.41 (−46.54, −19.90)	−43.58 (−60.64, −28.56)	−54.48 (−67.98, −38.26)	−67.47 (−78.54, −48.23)	
*N* (missing)	70 (3)	71 (2)	73 (0)	66 (7)	73 (0)	
NYHA Class, *n* (%)						<0.001
1	0	0	1 (1.4)	4 (5.6)	4 (5.5)	
2	45 (63.4)	59 (80.8)	68 (93.2)	68 (94.4)	69 (94.5)	
3	26 (36.6)	14 (19.2)	4 (5.5)	0	0	
*N* (missing)	71 (2)	73 (0)	73 (0)	72 (1)	73 (0)	

LAD, left atrial diameter; LAD, left atrial diameter; EF, ejection fraction; NT-proBNP, N-terminal pro-B-type natriuretic peptide; NYHA, New York Heart Association.

* The *P*-values for repeated measurement data analyzed using the generalized estimating equations represent the time effect.

**Table 3:** Effectiveness after 12 months of treatment in subgroup populations.

Follow-up	HFrEF (*n* = 14)	HFmrEF (*n* = 13)	HFpEF (*n* = 46)
LAD, mm, median (range)			
Baseline	49.00 (34.00, 59.00)	51.00 (44.00, 59.00)	50.00 (32.00, 76.00)
12 months	47.50 (32.00, 56.00)	48.00 (42.00, 56.00)	48.50 (33.00, 73.00)
Change	−1.50 (−4.00, 1.00)	−3.00 (−15.00, 2.00)	−2.00 (−7.00, 2.00)
E/e’, median (range)			
Baseline	11.25 (6.50, 24.80)	11.80 (6.80, 15.50)	10.00 (4.60, 19.20)
12 months	9.50 (5.10, 12.50)	8.90 (5.60, 11.80)	7.65 (4.10, 13.20)
Change	−2.65 (−18.70, 2.20)	−1.10 (−6.30, 0.70)	−2.70 (−13.40, 1.40)
*N* (missing)	12 (2)	13 (0)	38 (8)
EF, %, median (range)			
Baseline	34.50 (21.00, 39.00)	42.00 (40.00, 48.00)	63.00 (50.00, 71.00)
12 months	47.50 (30.00, 60.00)	48.00 (43.00, 60.00)	64.50 (52.00, 72.00)
Change	10.50 (2.00, 28.00)	5.00 (2.00, 18.00)	1.00 (−12.00, 7.00)
NT-proBNP, pg/mL, median (range)			
Baseline	1,477.42 (344.25, 15,302.00)	2,760.00 (331.20, 15,002.00)	1,415.50 (346.00, 4,692.00)
12 months	668.30 (137.60, 1,973.00)	880.00 (165.00, 6,573.00)	427.60 (91.60, 2,360.00)
Change, %	−54.55 (−91.73, −28.86)	−69.11 (−98.51, −19.36)	−68.82 (−93.44, 6.32)

HFrEF, heart failure with reduced ejection fraction; HFmrEF, heart failure with mildly reduced ejection fraction; HFpEF, heart failure with preserved ejection fraction; LAD, left atrial diameter; EF, ejection fraction; NT-proBNP, N-terminal pro-B-type natriuretic peptide.

NT-proBNP levels declined progressively throughout follow-up at 3 months of treatment (30.41%) and continued to decline. At the 12-month final follow-up, the median NT-proBNP decreased to 689 pg/mL, with a reduction of 67.47% compared with baseline (*P* < 0.001) (Figure 2C and Table 2). Similar reductions in NT-proBNP levels were observed across the three LVEF subgroups: 68.82% in HFpEF, 69.11% in HFmrEF and 54.55% in HFrEF (Table 3).

A progressive shift toward lower NYHA functional classes was observed during follow-up. All patients had symptoms at baseline (NYHA Class II: 58.9%; Class III: 41.1%). Over the course of treatment, the proportion of patients with NYHA Class III decreased, while the proportion with NYHA Class II increased. At 12 months, 100% of patients had cardiac function classified as NYHA Class I (5.5%) or II (94.5%) (Figure 2D and Table 2).

LVEF showed a modest increase in the overall cohort (Table 2), but the improvements were more pronounced in the HFrEF (from a median 34.5% to 47.5%, +10.5%) and HFmrEF (from a median of 42.0% to 48.0%, +5.0%) than in the HFpEF (from a median of 63.0% to 64.5%, +1.0%) subgroups (Table 3).

### Adverse events

No patients discontinued finerenone due to adverse events, suggesting that the drug was well tolerated in real-world patients with HF. Eight (11.0%) patients were re-admitted due to HF, and seven (9.6%) required an increase in diuretic dosage during follow-up.

The patients' blood pressure decreased steadily during treatment. As shown in Table 4, the systolic blood pressure and diastolic blood pressure decreased significantly from baseline (127.37 ± 16.47/73.21 ± 10.69 mmHg) to 1 month (121.01 ± 13.50/68.82 ± 8.34 mmHg, *P* < 0.001) and remained stable at all subsequent time points (at 12 months: 120.84 ± 12.67/68.78 ± 7.79 mmHg). Although the blood pressure reduction was statistically significant, no events requiring drug discontinuation due to symptomatic hypotension occurred during the study period. Only 4 patients (5.5%) had baseline SBP below 100 mmHg, and this number remained 4 at 12-month follow-up, with no reports of hypotension-related symptoms.

**Table 4:** Changes in safety measures in the total population.

Follow-up	1 month	3 months	6 months	9 months	12 months
SBP, mmHg					
Mean, SD	121.01 ± 13.50	122.65 ± 15.94	122.10 ± 13.61	122.39 ± 12.49	120.84 ± 12.67
*N* (missing)	73 (0)	71 (2)	73 (0)	67 (6)	73 (0)
DBP, mmHg					
Mean, SD	68.82 ± 8.34	69.15 ± 8.74	69.14 ± 8.86	68.67 ± 9.45	68.78 ± 7.79
*N* (missing)	73 (0)	71 (2)	73 (0)	67 (6)	73 (0)
sK^+^, mmol/L					
Median (range)	4.20 (3.48, 5.23)	4.10 (3.06, 4.90)	4.10 (3.06, 5.41)	4.15 (3.40, 4.90)	4.10 (3.31, 4.98)
*N* (missing)	72 (1)	71 (2)	73 (0)	66 (7)	73 (0)
eGFR, mL/min·1.73 m^2^					
Median (range)	65.30 (28.80, 111.81)	63.30 (29.70, 98.30)	67.00 (26.40, 102.00)	70.40 (26.50, 102.00)	70.10 (28.36, 109.39)
*N* (missing)	71 (2)	71 (2)	73 (0)	67 (6)	73 (0)

SBP, systolic blood pressure; DBP, diastolic blood pressure; sK+, serum potassium; eGFR, estimated glomerular filtration rate.

Serum potassium level was a key focus. As shown in Table 4, the baseline median serum potassium was 3.90 mmol/L. At 1 month, the median serum potassium increased to 4.20 mmol/L, a significant increase from baseline (*P* < 0.001). Thereafter, the serum potassium remained stable at a plateau of 4.10–4.15 mmol/L for the study period. Three patients (4.1%) had serum potassium levels ≥5.0 mmol/L. Importantly, no patients experienced severe hyperkalemia (>5.5 mmol/L). Episodes of hypokalemia (<3.5 mmol/L) occurred in four patients (5.5%) during follow-up. None of the potassium abnormalities led to treatment discontinuation or hospitalization.

Regarding renal function, as shown in Figure 3 and Table 4, the median eGFR showed a transient, slight decrease to 63.30 mL/min·1.73 m^2^ in the early stage of treatment (at 3 months), without significant differences from baseline (67.30 mL/min·1.73 m^2^) (*P* > 0.999). During treatment, eGFR showed a recovery trend, and the median eGFR increased to 70.10 mL/min·1.73 m^2^ at 12 months, which was significantly higher than at 3 months (*P* = 0.003) and did not differ significantly from baseline (*P* = 0.160). Specifically, an eGFR decline of >30% from baseline was observed in 3 (4.2%), 2 (2.8%), 5 (6.8%), 3 (4.5%), and 1 (1.4%) patients at 1, 3, 6, 9, and 12 months, respectively. Most of these declines were transient and resolved without permanent discontinuation of finerenone.

**Figure 3:**
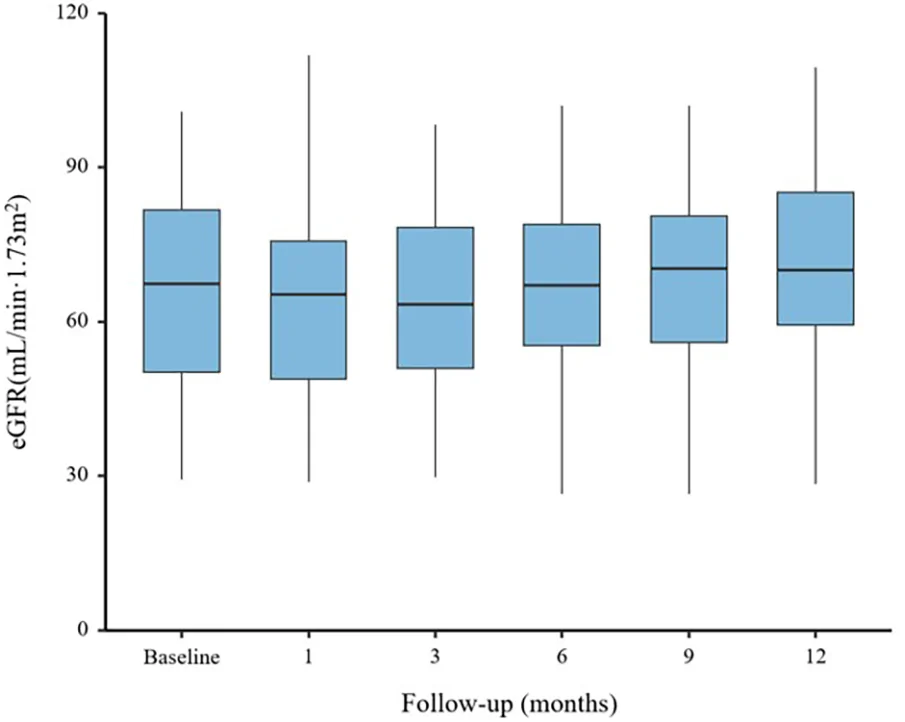
Longitudinal changes in estimated glomerular filtration rate (eGFR) over the 12-month follow-up.

## Discussion

During the 12-month follow-up period, favorable longitudinal changes in NT-proBNP, NYHA class, and echocardiographic parameters were observed in patients receiving finerenone. However, given the single-arm observational design and the inclusion of patients recovering from recent acute heart failure episodes, these findings should be interpreted as associations rather than evidence of a causal treatment effect.

The substantial reduction in NT-proBNP levels observed during follow-up warrants careful clinical interpretation. In our cohort, the median NT-proBNP decreased by 30.41% at 3 months and reached a 67.47% reduction at 12 months. Two key clinical factors likely contributed to the magnitude of this decline alongside finerenone initiation. First, 25 patients (34.2%) were enrolled during hospitalization for acute decompensated HF; in these patients, finerenone was started after intravenous loop diuretics had stabilized their volume status without requiring further diuretic escalation. Because natriuretic peptide levels surge acutely during decompensation, a substantial portion of the subsequent decline inevitably reflects natural post-discharge hemodynamic recovery following acute decongestion. Second, a subset of these hospitalized patients concurrently initiated SGLT2 inhibitors for the first time during the index admission, and background therapies (such as RASi and β-blockers) were maintained at stable doses throughout follow-up, with only loop diuretics adjusted as needed for volume management. Thus, the pronounced NT-proBNP reduction likely represents the combined effect of resolution from acute decompensation and synergistic unloading from concomitant SGLT2 inhibitor and background therapy, rather than the isolated pharmacological effect of finerenone. Biologically, this longitudinal decline in NT-proBNP reflects alleviated myocardial wall stress and attenuated neurohormonal activation (17). This finding is consistent across both the overall cohort and the HFpEF/HFmrEF subgroups, as corroborated by the FINEARTS-HF study (12, 31). ARTS-HF found finerenone non-inferior to eplerenone for achieving >30% NT-proBNP reduction over 90 days in HFrEF, supporting the concept that finerenone produces prognostically relevant natriuretic peptide lowering across the EF spectrum (18). The ARTS series of studies demonstrated that finerenone has an effect comparable to that of traditional MRAs on NT-proBNP, with better safety (18–20).

Notably, the improvement in the E/e' ratio was mainly observed in HFpEF/HFmrEF subgroups, reflecting reduced left ventricular filling pressure, which is similar to the improvement in diastolic function by spironolactone in the Aldo-DHF study (21). In addition, a pooled analysis of spironolactone in HFpEF (including Aldo-DHF and TOPCAT) confirmed favorable effects on echocardiographic structure and function, particularly E/e', further supporting the biological plausibility of the echocardiographic findings in the present study (22). Mechanistically, the cardioprotective effect of finerenone may be related to its unique pharmacological properties. Preclinical studies have shown that finerenone can alleviate cardiac diastolic dysfunction and improve cardiac perfusion in models of HFpEF (23). Compared with eplerenone, finerenone can more effectively improve left ventricular function in a rat HF model induced by coronary artery ligation (24).

In the present cohort, a shift toward lower NYHA functional classes was observed over 12 months, with all baseline Class III patients transitioning to Class I or II. However, in an unblinded, single-arm clinical setting, clinician-assessed NYHA functional class is inherently susceptible to observer and expectation bias, as well as survivorship bias among patients who completed the 12-month follow-up. While the randomized FINEARTS-HF trial demonstrated symptomatic benefits of finerenone using standardized, placebo-adjusted KCCQ scores (12, 25, 26), our unadjusted NYHA observations cannot be directly compared with blinded trial outcomes and should be viewed merely as descriptive clinical trajectories in routine practice. A retrospective study published in 2025 further confirmed that finerenone may have superior cardiovascular protective effects compared with traditional MRAs in patients with type 2 diabetes (27). This finding provides important evidence for the clinical application of finerenone, but further verification through prospective studies is still needed.

HFpEF accounts for more than half of patients with HF, and its 5-year survival rate is not significantly different from that of HFrEF (1). Currently, there are limited treatment options for HFpEF, and the emergence of finerenone brings new hope to this field. In the HFpEF subgroup, reductions in NT-proBNP levels and changes in NYHA class and echocardiographic parameters were observed during follow-up, findings corroborated by the Aldo-DHF study (21). The findings of the present study are broadly consistent in direction with observations from FINEARTS-HF, although direct comparisons are limited by important differences in study design and patient populations. Notably, the pronounced median LVEF increase (+10.5%) observed in our HFrEF subgroup should be interpreted with caution. Rather than representing a standalone pharmacological effect of finerenone, this improvement likely reflects the small subgroup sample size, functional recovery following recent acute decompensation, and the cumulative remodeling benefits of background quadruple therapy (such as ARNIs and SGLT2 inhibitors). For HFrEF, a clinical trial of finerenone in patients intolerant to spironolactone is ongoing (ClinicalTrials.gov NCT06033950). The present findings support further investigation of finerenone in patients with HFrEF, lending credibility to the ongoing trial.

The safety profiles of finerenone in this study are encouraging. Consistent with the results of randomized controlled trials such as FIDELIO-DKD, FIGARO-DKD, and FINEARTS-HF (10, 11, 28), finerenone showed good tolerability in the real world, with no patients discontinuing treatment due to adverse events. Notably, the incidence of hyperkalemia in this study was lower than in randomized controlled trials, which may be related to the high rate of SGLT2 inhibitor use (53.4%). SGLT2 inhibitors are known to have a potassium-excreting effect (29, 30), which may partially offset the potassium-elevating effect of finerenone. In addition, the trend of a transient, slight decrease in eGFR in the early stage followed by recovery is consistent with the adaptive regulation of intraglomerular pressure induced by MRA drugs. This hemodynamic change may be related to long-term renal protective effects (27). In the FIDELIO-DKD and FIGARO-DKD studies, finerenone exhibited clear renal protective effects (10, 11). Compared with traditional steroidal MRAs, finerenone has unique pharmacological advantages. Its nonsteroidal structure endows it with greater receptor selectivity and distinct tissue distribution (7). This pharmacological profile may contribute to the favorable tolerability observed in both clinical trials and routine practice.

## Limitations and future directions

This study has several limitations. First, the single-center observational design and relatively small sample size limit the generalizability of the findings. Second, the analysis included only patients who completed 12 months of follow-up, introducing the possibility of selection bias and survivorship bias. Third, approximately one-third of the cohort had recently been hospitalized for acute decompensated HF, and part of the observed improvement may reflect recovery following the acute episode and optimization of background therapy. Fourth, NYHA functional class assessment and echocardiographic measurements were not performed in a blinded fashion, and observer bias cannot be excluded. Finally, the absence of a control group prevents conclusions regarding the independent effects of finerenone.

## Conclusion

In this real-world observational cohort, treatment with finerenone was associated with favorable longitudinal changes in NT-proBNP levels, NYHA functional class, and selected echocardiographic parameters, together with an acceptable safety profile. Given the observational design and lack of a control group, causal inferences cannot be made. Larger prospective controlled studies are needed to confirm these findings.

## Data Availability

The raw data supporting the conclusions of this article will be made available by the authors, without undue reservation.
